# Construction of Aptamer-Based Nanobiosensor for Breast Cancer Biomarkers Detection Utilizing g-C_3_N_4_/Magnetic Nano-Structure

**DOI:** 10.3390/bios12110921

**Published:** 2022-10-25

**Authors:** Mehrab Pourmadadi, Fatemeh Yazdian, Sohrabali Ghorbanian, Amin Shamsabadipour, Elham Khandel, Hamid Rashedi, Abbas Rahdar, Ana M. Díez-Pascual

**Affiliations:** 1 Department of Biotechnology, School of Chemical Engineering, College of Engineering, University of Tehran, Tehran 11155-4563, Iran; 2 Department of Life Science Engineering, Faculty of New Science and Technology, University of Tehran, Tehran 14166-34793, Iran; 3 School of Chemical Engineering, College of Engineering, University of Tehran, Tehran 14166-34793, Iran; 4 Department of Physics, Faculty of Science, University of Zabol, Zabol 538-98615, Iran; 5 Universidad de Alcalá, Facultad de Ciencias, Departamento de Química Analítica, Química Física e Ingeniería Química, Ctra. Madrid-Barcelona, Km. 33.6, 28805 Alcalá de Henares, Madrid, Spain

**Keywords:** aptamer, nanomaterial, g-C_3_N_4_, biomarker, biosensor, breast cancer

## Abstract

An electrochemical aptasensor has been developed to determine breast cancer biomarkers (CA 15-3). Aptamer chains were immobilized on the surface of the electrode by g-C_3_N_4_/Fe_3_O_4_ nanoparticles, which increased the conductivity and active surface area of the electrode. X-ray diffraction analysis (XRD), Fourier-transformed infrared spectroscopy (FTIR), and transmission electron microscopy (TEM) measurements have been carried out to characterize the nanomaterials. Cyclic voltammetry, square wave voltammetry, and electrochemical impedance spectroscopy have been used to characterize the developed electrode. The results demonstrate that the modified electrode has better selectivity for CA 15-3 compared to other biological molecules. It has a good electrochemical response to CA 15-3 with a detection limit of 0.2 UmL^−1^ and a linear response between 1 and 9 UmL^−1^. It has been used as a label-free sensor in potassium ferrocyanide medium and as methylene blue-labeled in phosphate buffer medium. This electrode was successfully applied to analyze the serum of diseased and healthy individuals, which corroborates its high potential for biosensing applications, especially for the diagnosis of breast cancer.

## 1. Introduction

Breast cancer is common amongst women, and early cancer detection can lead to successful therapies and thus reduce mortality from this cancer [1]. Cancer is recognized by biomarkers, which are also a means of detecting the stage or intensity of this illness [2]. Cancer biomarkers are proteins produced by the body or tumor in a person with cancer [3]. Cancer Antigen 15-3 (CA 15-3) and Carcino Embryonic Antigen (CEA) are two types of biomarkers of breast cancer, though the former is used more often to detect this cancer owed to its better clinical specificity [4]. Concentrations of CA 15-3 in the blood of patients with breast cancer increase to 10% in stage Ⅰ, to 20% in stage Ⅱ, to 40% in stage Ⅲ and to 75% in stage Ⅳ [5]. 

Some common methods for detecting cancer cells require advanced machines and skilled people, which increases the cost [6]. Additionally, due to the separation of infectious agents and subsequent cultures, these types of diagnoses are very time-consuming [6]. The most common way of detecting cancer cells is based on antigen–antibody interactions [7]. Antibodies used in cancer treatment are obtained from sacrificing animals in very expensive and uncontrollable environmental conditions [8].

A biosensor is a device for measuring an analyte through a biological interaction [9]. Biosensors can be used to detect hormones, drugs, contaminants, heavy metals, etc. [5]. The biosensor consists of three parts: bioreceptor, transducer, and detector. The bioreceptor specifically recognizes the analyte, since the interactions between the bioreceptor and the analyte are very specific. The role of the transducer is to transform these interactions into an electrical signal and that of the detector is to amplify the signal provided by the transducer to make it readable [9]. Antibodies, enzymes, cells, and aptamers are different types of receptors [10]. In this work, an aptamer has been selected because of its stability, selectivity, and cost.

Aptamers are molecules identified during an in vitro selection process. In this process, called SELEX (Systematic Evolution Of Ligands by Exponential Enrichment), RNA sequences that specifically bind to the target molecules are separated [11]. Therefore, the specific aptamer can be identified to bind to a target molecule [11]. They are widely used in the biomedical field including diagnostics and novel drug delivery [6]. Aptamers can act as receptors in sensors [6]; they have better thermal stability than antibodies, are more resistant to organic solvents, and have lower immunogenicity. Additionally, due to their smaller size, they can be easily synthesized in large quantities [6].

Nanobiosensors are sensors constructed using nanosized materials, that is, with one dimension between 1 and 100 nanometers [9]. The application of nanomaterials in biosensors improves measurement accuracy, therefore resulting in a sensor with a lower detection limit. Nanomaterials have an extremely high surface-to-volume ratio, which causes a very strong interaction between the sensor and the analyte [12]. Currently, nanomaterials are widely used in diagnostic and therapeutic fields. This includes the use of nanomaterials to minimize chemotherapy problems [13]. Since chemotherapy faces issues such as low drug loading efficiency and uncontrolled drug distribution, researchers are trying to reduce the destructive effects of the drug before it reaches the target organs by designing targeted and stable nanocarriers with specific drug release capacities [13]. This work used graphitic carbon nitride and magnetic nanoparticles as a nanostructure. Graphitic carbon nitride (g-C_3_N_4_) has attracted much attention over recent years [14]. It has a 2D structure in which the chemical Heptazine is attached to the tertiary amine groups [15,16]. This is a metal-free semiconductor nanoparticle that is chemically and thermally stable. It also exhibits good catalytic, optical, and electronic properties [17]. It is applicable in sensing, drug delivery [18], imaging, and environmental fields [19].

Electrochemical nanobiosensors have received considerable attention because of their features such as low cost, ease of use, and ultrasensitive detection [12]. In electrochemical biosensors, electrochemical techniques are used to analyze biological processes. These types of sensors can be used in processes that exchange ions or electrons between the bioreceptor and the target analyte [20]. 

Fe_3_O_4_ nanoparticle is a magnetic material with a wide range of applications due to features such as stability, easy synthesis, high surface energy, high surface area, and acting as electron transfer paths [21]. Magnetic nanoparticles (MNPs) facilitate the electron transfer between the electrolyte medium and the electrodes [20]. Thus, adding Fe_3_O_4_ to g-C_3_N_4_ sheets can enhance the electro conductivity, which in turn can lead to higher sensitivity (lower *LOD*) and higher precision as well as to improved structural stability of the synthesized g-C_3_N_4_ and the whole nanobiosensor.

In the current study, an aptasensor for the early detection of breast cancer using g-C_3_N_4_/Fe_3_O_4_ as a biosensor substrate has been developed for the first time [22]. For this purpose, firstly, g-C_3_N_4_/Fe_3_O_4_ was synthesized and then the aptamer was incubated with the mentioned nanocomposite and subsequently attached to the electrode surface. Then, the synthesized nanocomposites were studied by X-ray diffraction (XRD) and Fourier-transformed infrared spectroscopy (FTIR). Calibration and selectivity evaluation of the nanosensor was performed by electrochemical tests in different modes of cyclic voltammetry, square wave, and impedance measurements in a potassium ferrocyanide medium K_4_(Fe(CN)_6_). After performing the desired electrochemical tests in K_4_(Fe(CN)_6_), an attempt was made to reduce the damage of performing experiments in a potassium ferrocyanide medium by labeling the working electrode with methylene blue. Finally, the fabricated nanobiosensor was validated using real samples of cancer serum.

The research reported herein is crucial for the precise study and to collect of the necessary information about electrochemical methods for the recognition of breast cancer biomarkers (CA 15-3), which are caused by an abnormal increase in the specific antigen of this cancer. High specificity and sensitivity, very simple operation, and low cost are among the main advantages of the device developed in this research work. Additionally, the use of nanotechnology and the synthesis of nanomaterials to improve nanocomposite performance and selection differ from previous research works, and the use of magnetic nanomaterials along with an aptamer in the receptor section are among the innovations of the present study.

## 2. Experimental

### 2.1. Materials and Methods

Urea, FeCl_3_·6H_2_O, FeCl_2_·4H_2_O, ammonia, glutaraldehyde, fetal bovine serum (FBS), glucose and bovine serum albumin (BSA), potassium ferrocyanide, and methylene blue were all acquired from Sigma Aldrich (Germany http://www.sigmaaldrich.com (accessed on 20 January 2021), Cancer Antigen 15-3 (CA 15-3) was purchased from Monobind Inc. (http://www.monobind.com (accessed on 20 January 2021). Prostate-Specific Antigen (PSA) was purchased from Antibody Science Company (Iran) (http://www.padtan.com (accessed on 18 January 2021) and was employed as received. All the solutions were prepared in deionized water. Morphological characterization was carried out via transmission electron microscopy (TEM, Philips Tecnai G220, operating at 120 kV, https://www.jeolusa.com (accessed on 18 April 2021). The crystalline structure was investigated via X-ray diffraction (XRD) analysis, which was performed on an X-ray diffractometer (STOE, Germany https://www.stoe.com (accessed on 18 April 2021) with a Cu-Kα as a radiation source, and FT-IR measurements were performed with a spectrometer (Thermo Inc., Waltham, MA, USA; https://www.thermofisher.com (accessed on 20 April 2021).

The CA 15-3 aptamer was fabricated by bio basic inc. (Canada, https://www.biobasic.com (accessed on 30 April 2021) with the following sequence: (GAAGTGAATATGACAGATCACAACT). The freeze-dried aptamer powder was diluted by PBS at pH 7.4 and maintained at 4 °C. The electrochemical experiments were performed with a potentiostat/galvanostat device designed by Ivium technologies, (Netherlands https://www.ivium.com (accessed on 18 April 2021) in ferricyanide redox probe 0.2 mM and PBS 0.1 M. A three-electrode system composed of a glassy carbon electrode with the obtained diameter of 2 mm for working electrode [10], a Platinum electrode, and an Ag/AgCl electrode as counter and reference, respectively (Detectco https://www.detectco.com (accessed on 18 May 2021). 

### 2.2. Synthesis of g-C_3_N_4_

Five g of urea was placed in a porcelain crucible and put in an oven to reach 450–550 °C at a heating rate of 3–5 °C.min^−1^. Then, it was kept at that temperature for 3–4 h and subsequently cooled to room temperature [23,24].

### 2.3. Synthesis of g-C_3_N_4_/Fe_3_O_4_ Nanocomposite

For the synthesis of g-C_3_N_4_/Fe_3_O_4_, 100 mg of the pre-prepared g-C_3_N_4_ powder was added to 50 mL distilled water in a sonication bath for 10 min and then 90 mg of FeCl_2_·4H_2_O was dropped into the mixture and sonicated in a bath at 30 °C for 10 min. Then, ammonia solution (25% *w*/*w*) was added to increase the pH to 10, and the mixture was then stirred at 80 °C for 30 min [21].

### 2.4. Incubation and Preparing the g-C_3_N_4_/Fe_3_O_4_/Apt

Following the aforementioned, 98 µL of g-C_3_N_4_/Fe_3_O_4_ nanocomposite (4 mg mL^−1^) was mixed with 5 µL of glutaraldehyde, and after stirring for 2 h, 2 µL of aptamer 100 µM was added and refrigerated at 4 °C for 24 h.

### 2.5. Electrode Preparation Procedure

First, the electrode surface was polished with alumina powder-5µm (Al_2_O_3_) to ensure that the electrode surface is clean. Cyclic Voltammetry (CV, potential range from −0.5 to +0.7 V with scan rate 50 mVs^−1^), Square Wave Voltammetry (SWV, −0.4 to 1.0 V with amplitude 10 mV) and Electrochemical Impedance Spectroscopy (EIS, 32 frequency points, 0.01 to 100,000 Hz) tests were performed in potassium ferrocyanide medium 0.2 mM, and then 4 μL g-C_3_N_4_ was dropped on the electrode surface until it was dried under normal lamplight. For all electrochemical tests, 4 μL of the synthesized nanomaterials were dropped on the surface of the modified electrode for a preset time, and afterward, the electrode was washed with PBS solution (pH = 7.4) to release unbonded substances at the surface of the electrode. Finally, the modified electrode was prepared for the electrochemical assessment.

### 2.6. Time Profile Incubation/Interaction Time between the Aptamer and the Analyte

To obtain a suitable time for detection of CA 15-3 by nanoprobe, diverse incubation times were utilized for 10 μL CA 15-3 at a concentration of 50 UmL^−1^. The response of the nanoprobe becomes stable after 35 min, thereby, this time has been chosen for CA 15-3 assessment by the nanoprobe.

### 2.7. Electrochemical Characterization of Nanobiosensor

After electrode preparation, CV, SWV, and EIS tests were carried out. For such purpose, the electrode surface was first cleaned, and afterward, 4 μL of g-C_3_N_4_/Fe_3_O_4_ solution was dropped on the electrode surface and then dried under normal lamplight. Four μL of g-C_3_N_4_/Fe_3_O_4_/Apt solution, which was previously incubated in a refrigerator at 4 °C for 24 h, was dropped on the electrode surface to ensure aptamer binding to the C_3_N_4_/Fe_3_O_4_ surface and electrochemical modifications. Then, 10 µL of the analyte (CA 15-3), 50 U·mg^−1^, were dropped on the electrode surface containing g-C_3_N_4_/Fe_3_O_4_/Apt and incubated by placing the cap on the electrode surface at room temperature. After 35 min, the unbound analyte to aptamer was washed by PBS at pH 7.4 and the above tests were performed.

### 2.8. Concentration Test of Biosensor

After preparation of the electrode according to the mentioned protocol, 4 μL of incubated g-C_3_N_4_/Fe_3_O_4_/Apt nanocomposite was dropped on the electrode surface and placed directly under a normal lamplight to dry and CV, SWV, and EIS tests were performed with the same parameters as before. Then, without clearing the electrode surface, 10 μL of the lowest CA 15-3 (10 UmL^−1^) concentration was dropped on the electrode surface and incubated for 35 min by placing the cap on the electrode surface to prevent the sample from drying. After 35 min, the residual amount of unbound CA 15-3 was rinsed with 100 mM of PBS at physiological pH conditions, and CV, SWV, and EIS tests were performed again. Then, without clearing the electrode surface, all the above tests were performed from low to high CA 15-3 concentrations (30, 50, 70, and 90 UmL^−1^) using the same parameters as indicated above. 

### 2.9. Concentration Test of Label-Based Biosensor

After electrode preparation, it was placed upside down in 2.5 mM methylene blue solution for 45 min. The methylene blue that was not linked to the aptamer backbone was washed with 100 mM PBS pH = 7.4. Then CV (potential range from −0.4 to 0 V with scan rate 50 mVs^−1^) and SWV (−0.4 to 1.0 V with amplitude 10 mV) tests were performed in PBS medium. Next, 10 μL of the lowest CA 15-3 concentration (1 UmL^−1^) was dropped on the electrode surface and incubated for 35 min by placing the cap on the electrode surface to prevent the sample from drying. After 35 min, the residual amount of unbound CA 15-3 was washed with 100 mM PBS pH = 7.4. Then CV and SWV tests were performed using the same parameters as mentioned before. Likewise, it was applied to consecutive CA 15-3 concentrations (3, 5, 7, and 9 UmL^−1^). 

### 2.10. Stability Test of Label-Based Biosensor

Due to the importance of the stability of the designed biosensor, 50 cycles of CV tests (potential range from −0.4 to 0 V with scan rate 50 mVs^−1^) were taken for the labeled nanoprobe.

### 2.11. Diffusion Control Test of Label-Based Biosensor

To evaluate the quality of the biosensor, CV tests were carried out at scan rates of 25, 50, 75, 100, 125, 150, 200, 250, and 300 mVs^−1^ (the potential range of −0.4 to 0 V) for the labeled nanoprobe. 

### 2.12. Selectivity Test of the Electrochemical Biosensor to CA 15-3 Detection

The selectivity of the biosensor was assessed by testing it for other chemical compounds that may be present in the blood. To prove that this biosensor is highly selective to CA 15-3, SWV was performed in 0.2 mM potassium ferrocyanide in the potential range from −0.4 to 1 V for five substances: glucose, FBS, BSA, PSA, and CA 15-3 at concentrations of 90 mg dL^−1^, 5 ng mL^−1^, 7 μg mL^−1^, 2.5 pg mL^−1^, and 5 UmL^−1^, respectively, and the electrochemical signals generated by the samples were compared.

### 2.13. Real Sample Analysis

Six serum samples consisting of 3 patients and 3 healthy individuals were used. Ten µL of the real sample was employed as an analyte on the surface of the electrodes, followed by incubation with the aptamer for 35 min, similar to the selectivity procedure. Afterward, residual amounts were rinsed by adding PBS at pH 7.4.

## 3. Results and Discussion

### 3.1. Morphological Assessment

**Figure 1:**
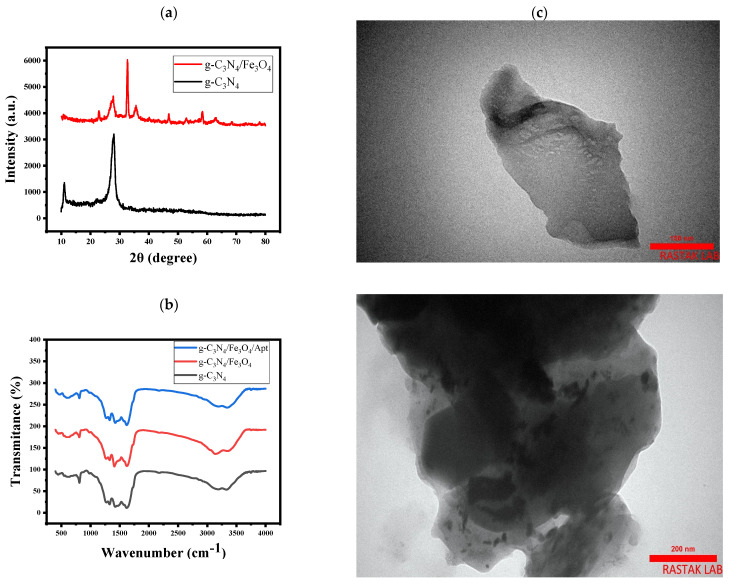
(**a**) X-ray pattern of g-C_3_N_4_ and g-C_3_N_4_/Fe_3_O_4_. (**b**) FTIR spectra of g-C_3_N_4_ and g-C_3_N_4_/Fe_3_O_4_ and g-C_3_N_4_/Fe_3_O_4_/Apt. (**c**) TEM image of g-C_3_N_4_ and g-C_3_N_4_/Fe_3_O_4._

The crystalline structure of the developed samples was investigated by XRD (Figure 1a). The XRD pattern of g-C_3_N_4_ shows a peak at 2θ = 10.95° and a sharp peak at 2θ = 27.75°, which arise from the reflection of the (100) and (002) planes of g-C_3_N_4_ sheets [25], corresponding to the inter-layer structural packing and the interplanar stacking peaks of aromatic systems, respectively [26]. These results are consistent with a study conducted in 2018 by Mousavi et al. [27], who synthesized novel ternary g-C_3_N_4_/Fe_3_O_4_/MnWO_4_ nanocomposites for environmental purposes and used the XRD test to evaluate the properties of the synthesized nanocomposites. They also reported peaks at 2θ = 13.1° and 2θ = 27.4°, which are almost identical to the values obtained in the current study. Additionally, Wang et al. carried out the synthesis of g-C_3_N_4_/Fe_3_O_4_ nanocomposites and investigated their application as a new adsorbent for solid-phase extraction of polycyclic aromatic hydrocarbons in water samples [21]. A peak at 2θ = 27.41° was reported for g-C_3_N_4_ sheets, in agreement with the results of this work. In the XRD spectrum of g-C_3_N_4_/Fe_3_O_4_, the diffraction peaks of both g-C_3_N_4_ and Fe_3_O_4_ can be observed. Two typical diffraction peaks corresponding to the (100) and (002) diffraction planes derived from g-C_3_N_4_ are found, and the other diffraction peaks can be indexed to the cubic structure of Fe_3_O_4_ (JCPDS 19-0629) [28]. The peaks at 32.7°, 35.6°, 46.8°, 52.8°, 58.4°, and 63.1° belong to the (220), (311), (400), (422), (511), and (440) planes of cubic Fe_3_O_4_ [25].

The FTIR test was used to obtain insight about the surface functional groups of the composite components and how they bind as well as to confirm the binding of the aptamer to g-C_3_N_4_/Fe_3_O_4_ nanocomposite. Figure 1b shows the FTIR analysis of g-C_3_N_4_ and g-C_3_N_4_/Fe_3_O_4_ and g-C_3_N_4_/Fe_3_O_4_/Apt nanostructures. Wang et al., in a study performed in 2015, reported that the signal at 606 cm^−1^ corresponds to the Fe-O band [21], while the peak at 807 cm^−1^ was attributed to the typical breathing vibration of triazine units in the skeletal structure of g-C_3_N_4_ [21,25]. In the absorption range of 1267–1626 cm^−1^, the peaks at 1267, 1326, 1408, and 1626 cm^−1^ were assigned to either trigonal C–N(–C)–C (full condensation) or bridging C–NH–C (partial condensation) units [19]. This strong range is related to the stretching vibrations of CN heterocyclic [27]. The wide absorption peak at 3000–3400 cm^−1^ can be attributed to the O-H stretching vibration of surface adsorbed water and the N-H stretching vibration of residual NH_2_ bonded to the sp^2^ hybridized carbon or NH groups at the defect sites of the aromatic ring [29]. Moreover, the morphology of the samples was assessed by TEM (Figure 1c), which revealed that the synthesized sheet-like g-C_3_N_4_ and g-C_3_N_4_ were completely covered by well-dispersed Fe_3_O_4_ magnetic nanoparticles. The wrapping by Fe_3_O_4_ nanoparticles improved the conductivity of the g-C_3_N_4_ and also created a more appropriate substructure for aptamer arrangement.

### 3.2. Electrochemical Features of the Modified Electrode

**Figure 2:**
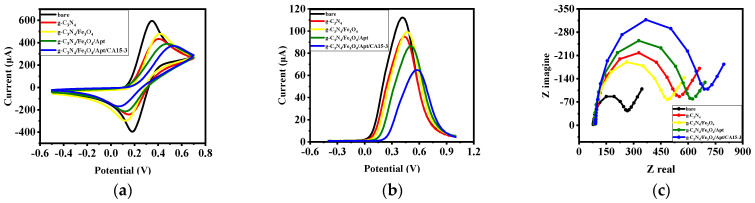
Electrochemical characterization of the developed nanobiosensor by (**a**) CV analysis, (**b**) SWV analysis and (**c**) EIS analysis of the bare electrode and each step of the preparation of the modified electrodes in K4[Fe(CN)6] −3−4 (0.2 mM).

The electrode modification process was investigated using electrochemical methods. Figure 2a shows the CV electrode curves after each modification step. According to the figure, each CV curve has a redox peak, due to the K4[Fe(CN)6] −3−4 reaction at that potential. The bare electrode had relatively high maximum oxidation and reduction current peaks. Modification of the electrode surface with g-C_3_N_4_ decreased the maximum oxidation current peaks. After modifying the electrode surface with g-C_3_N_4_/Fe_3_O_4_, the current peaks increased due to the increase in the active surface area of the electrode and the improvement in conductivity. By attaching aptamer chains to the g-C_3_N_4_/Fe_3_O_4_ surface, some active sites were blocked, reducing the current peaks of CV curves. In addition, after the folding process between CA 15-3 and aptamer chains, the current peaks of the K4[Fe(CN)6] −3−4 reaction decreased again. SWV voltammograms of the modified electrode at each step are shown in Figure 2b. As can be observed, the results obtained from this electrochemical method are consistent with the results of the CV technique. EIS is an electrochemical technique that provides useful information about charge transfer at the electrode contact surface. Nyquist diagrams for each stage of the electrode modification are shown in Figure 2c. As shown, each diagram has a semicircle corresponding to the charge transfer resistance (R_ct_) of the reaction [Fe(CN)6] −3−4, which appears in the diameter of the semicircles. Modification of the electrode surface with different materials changes the resistance of the electrode surface and therefore, the diameter of the semicircle increases in all steps except the g-C_3_N_4_/Fe_3_O_4_ modification step, which decreases the diameter of Nyquist diagrams due to the increase in conductivity and in the electrode surface in this stage.

### 3.3. Time Detection Analysis

**Figure 3:**
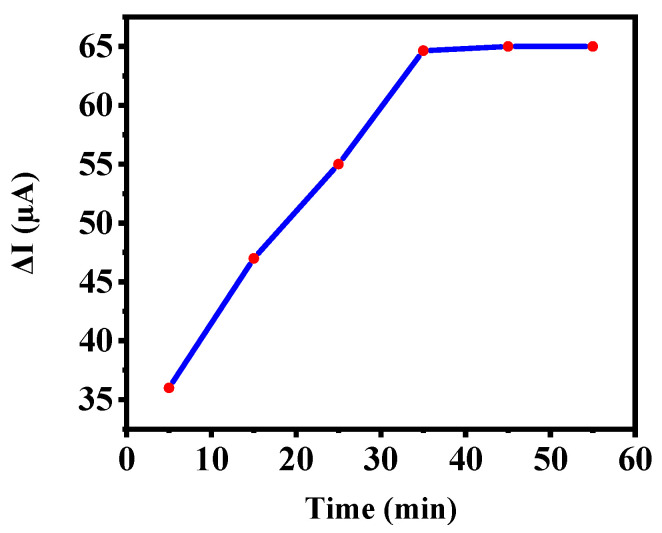
Time profile of aptamer/nanobiosensor interaction based on SWV techniques at different incubation times.

To find the optimal time to detect CA 15-3 by the nanoprobe, different incubation times with CA 15-3 at a concentration of 50 UmL^−1^ were used. The electrode time profile is shown in Figure 3. According to the figure, protein uptake was negligible for the first 5 min, indicating that the uptake rate was very low. With increasing time up to 35 min, the adsorption rate increases, but from 35 to 45 min, the adsorption rate is very low, which reveals that time does not affect analyte adsorption for periods larger than 35 min, and that the electrode response stabilizes. Therefore, the optimal time to determine CA 15-3 is 35 min. 

### 3.4. Stability Assessment of the Nanobiosensor

**Figure 4:**
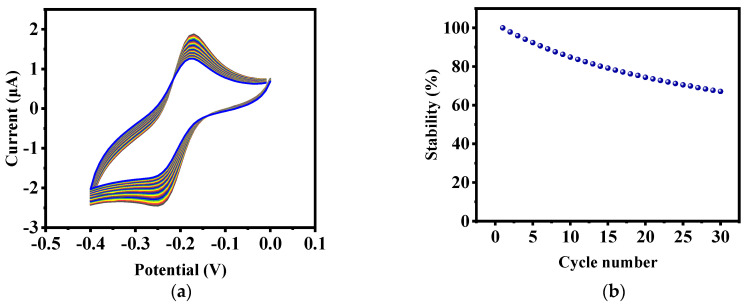
(**a**) Stability test of the electrode labeled by methylene blue. (**b**) Percentage of electrochemical stability of methylene blue electrode.

Due to the importance of the stability of the designed biosensor, 50 cycles of CV tests were taken in the potential range of −0.4 to 0, and the results are shown in Figure 4a. As can be observed, as the number of experiments increases, the CV curves hardly change, thus corroborating the high stability of the designed biosensor. Figure 4b shows that this nanobiosensor keeps about 70% stability after 30 cycles.

### 3.5. Electrochemical Characterization of the Nanobiosensor

**Figure 5:**
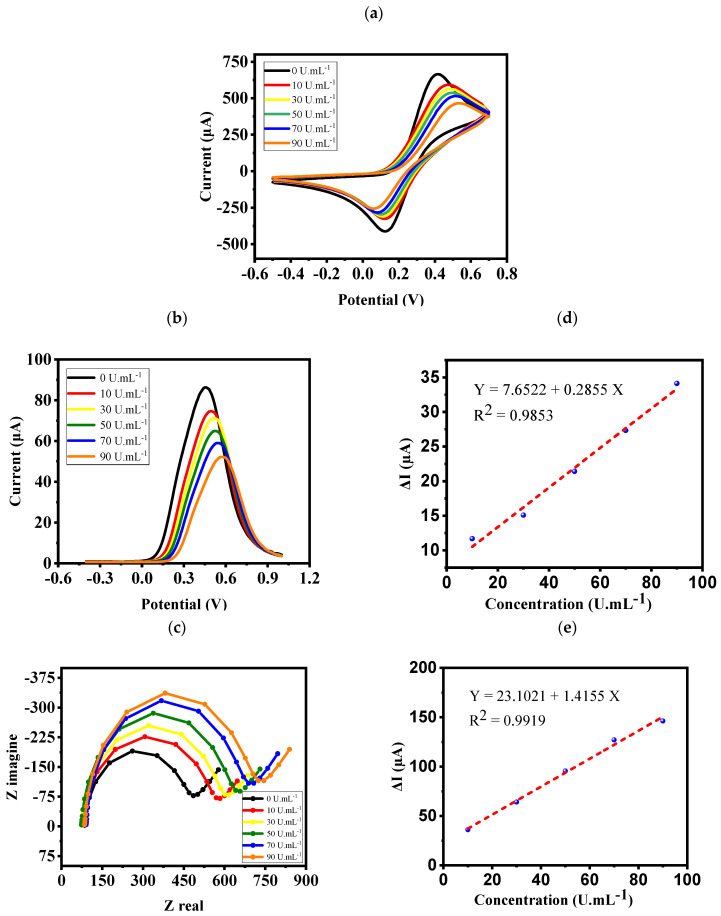
(**a**) CV analysis, (**b**) SWV voltammograms, and (**c**) Nyquist diagrams of the electrode in K4[Fe(CN)6] −3−4 (0.2 mM) media at different concentrations of CA 15-3 ranging from 0 to 90 UmL^−1^. (**d**,**e**) CV and SWV linear responses of the nanoprobe to different concentrations of CA 15-3.

Figure 5a,b show CV analysis and SWV voltammograms, respectively, of the nanoprobe electrode in [Fe(CN)6] −3−4 at different concentrations of CA 15-3. According to the figures, the binding of CA 15-3 to the surface of the electrode through the interaction of CA 15-3/aptamer prevents the reaction of [Fe(CN)6] −3−4 on the electrode surface, and thus the peak currents decrease. This reduction of the peak current occurs following a linear behavior. The linear response (from 10 to 90 UmL^−1^) of CA 15-3 nanoprobe versus concentration is displayed in Figure 5d. The Nyquist diagrams of the nanoprobe electrode in [Fe(CN)6] −3−4 are shown in Figure 5c. As the concentration of CA 15-3 at the electrode surface increases and the folding process progresses, the resistance of the electrode surface (R_ct_) increases. In addition, when these molecules are connected to the surface of the electrode, the value of R_ct_ increases, which appears in the diameter of the semicircles. Similar to SWV, the nanoprobe has a linear response with high and acceptable R^2^ for different concentrations of CA 15-3 (Figure 5e). The detection limit is another characteristic of the biosensor that can be calculated from the equation below [30]:

(1) $$LOD=\frac{3\;S_{B}}{M}$$

where *S_B_* is the standard deviation of the bare surface and m is the slope of the calibration diagram. The *LOD* value obtained using Equation (1) was found to be 0.2 UmL^−1^ based on SWV data.

**Figure 6:**
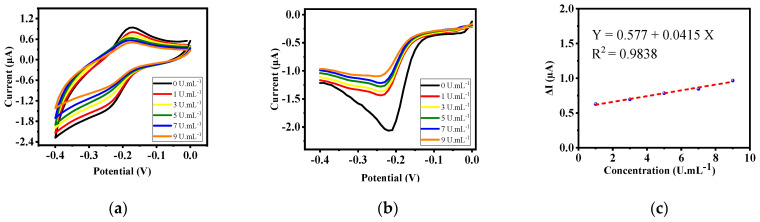
(**a**) CV and (**b**) SWV test results at (**c**) various concentrations of CA 15-3 with the labeled electrode by methylene blue.

Even though potassium ferrocyanide has a good electrochemical signal, it is hazardous. So, an alternative substance was selected as a redox agent. One of the alternatives for this purpose is methylene blue. The toxicity of this material is very low and it can be used in labeled form, also this material has optical properties. The reason for placing the electrode upside down in methylene blue instead of pouring it on the electrode surface is to eliminate the effects of gravity and thus to attain higher accuracy. Figure 6a,b show the CV curves and SWV voltammograms of the methylene blue-labeled electrode, respectively. The results show a decrease in the electrochemical peak with increasing sample concentration and a linear response (from 1 to 9 UmL^−1^) with a high and acceptable R² for all the tests and all the concentrations used (Figure 6c).

**Figure 7:**
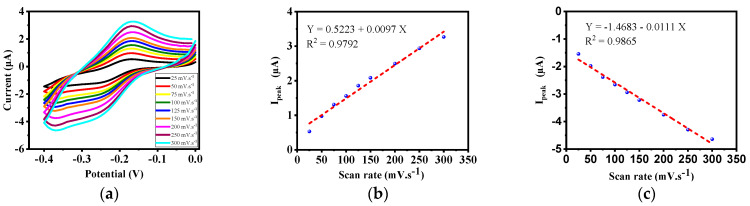
(**a**) Diffusion control test of the electrode labeled by methylene blue. (**b**) Calibration curve of diffusion control test of the electrode labeled by methylene blue in the reduction path. (**c**) Calibration curve of diffusion control test of the electrode labeled by methylene blue in the oxidation path.

The results of the diffusion control test are presented in Figure 7a. According to the figure, as the scan rate increases, the maximum current increases, which indicates that the designed biosensor has an acceptable performance. Additionally, according to the calibration results of the electrode labeled with methylene blue (Figure 7b,c), it is found that the current is linear in both directions, and that the designed sensor has a proper performance.

**Figure 8:**
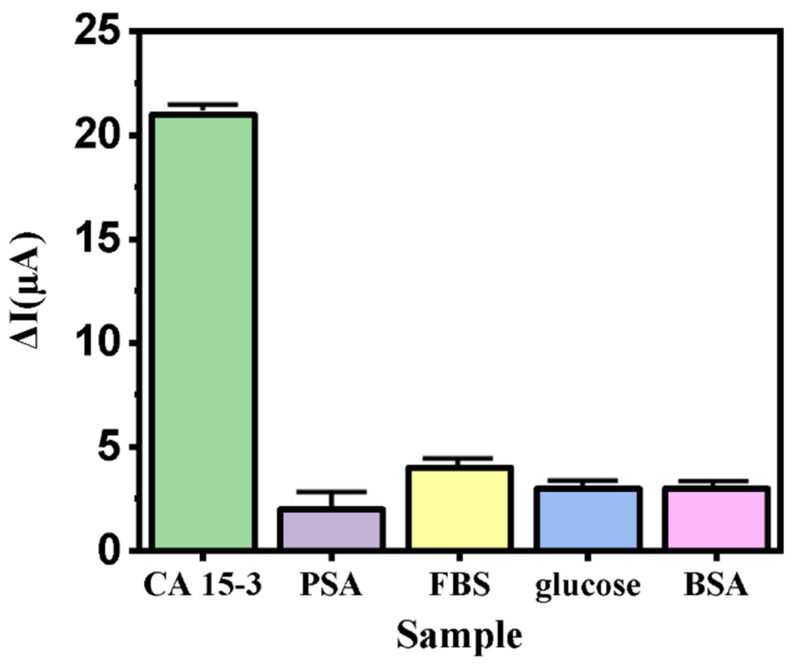
Comparison of nanoprobe electrode signal for CA 15-3 (5 UmL^−1^) glucose (90 mg dL^−1^), PSA (2.5 pg mL^−1^), FBS (5 ng mL^−1^), BSA (7 μg mL^−1^).

Figure 8 shows the selectivity of the nanoprobes against some biological molecules. As can be observed, the nanoprobe exhibits a stronger response to very low concentrations of CA 15-3 than to other materials. This confirms the high selectivity of the biosensor to the CA 15-3 analyte. The selectivity of the nanobiosensor in a biomimetic situation for some regular compounds in blood was investigated, indicating a higher affinity to CA 15-3 with a lower *LOD* compared with other elements. This indicates the great potential of the developed nanobiosensor to be applied in in vivo situations.

**Figure 9:**
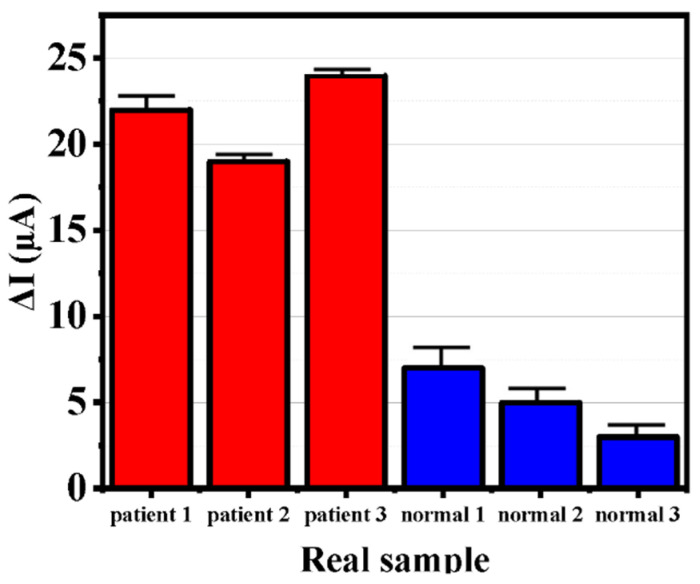
Real sample test results based on SWV techniques.

The purpose of such nanobiotechnology studies is to use these nanobiosensors in the medical field. The results of SWV voltammograms of 3 normal serum samples and 3 patient serum samples are shown in Figure 9. According to the figure, there is a significant difference between 3 patient serum samples and 3 normal serum samples, which confirms that the sensor was able to respond well to the desired analysis. The results show that following the approach developed herein, electrochemical nanoprobes can be used for medical purposes. The current difference is less than 8 µA for all healthy serum samples and more than 17 µA for all patient serum samples. That difference is quite clear. Therefore, it can be concluded that this biosensor can be used to diagnose breast cancer.

The prepared nanobiosensor has been applied to real serum samples to detect CA 15-3; however, compared with other conventional methods such as the use of particular kits, it is worth considering some issues. Most of the ELISA kits usually employed for detecting CA 15-3 or other conventional methods are not accurate (i.e., have large error bars [31,32]), cannot be reused for other samples and are quite expensive compared to the nanobiosensor developed herein. Moreover, it should be noticed that the developed nanobiosensor, due to its low *LOD* (0.2 UmL^−1^), is more sensitivity and can be applied effectively to detect lower concentrations of serum compared to ELISA kits (sensitivity of 4 UmL^−1^) or other conventional methods.

Table 1 provides a comparison between the developed aptasensor with other studies for CA 15.3 detection. As can be observed, the provided g-C_3_N_4_/Fe_3_O_4_ electrochemical electrode can be an appropriate nanoprobe for biosensing applications.

## 4. Conclusions

Due to the importance of early detection of cancers that can lead to successful treatment, this study proposed a nanobiosensor that can be applied to detect the early stage of breast cancer with a low *LOD* and a high sensitivity. The developed electrochemical nanoprobe electrode showed better selectivity over CA 15-3 compared to other biological materials. Decoration of the electrode with g-C_3_N_4_/Fe_3_O_4_ led to a rapid aptamer immobilization on the electrode surface and increased the electrochemical conductivity. The stability test of the methylene blue-labeled electrode showed that the electrode kept about 70% stability after 50 cycles and that its response to different CA 15-3 concentrations was linear. All the electrochemical results and data were statistically acceptable, with a high correlation coefficient. A wide linear response in the range of 1–9 UmL^−1^ and a low *LOD* of 0.2 UmL^−1^ was attained. Real samples of healthy and diseased patients were also successfully tested, which demonstrates the suitability of the designed nanoprobe for biosensing applications, especially for breast cancer diagnosis.

## Figures and Tables

**Table 1:** Comparison of the provided biosensor with other studies for CA 15.3 detection.

Technique	Active Components	Linear Response	*LOD*	Reference
Electrochemical	Pyrrole	1.44–13.2 UmL^−1^	1.07 UmL^−1^	[33]
Electrochemical	gold nanosphere assembled onto thiolated graphene quantum dots (GQD/Cys/AuNPs)	0.16–125 UmL^−1^	0.11 UmL^−1^	[34]
Electrochemical immunosensor	silver nanoparticles-reduced graphene oxide (Ag/RGO)	15–125 UmL^−1^	15 UmL^−1^	[35]
Electrochemical immunosensor	reduced graphene oxide (RGO) and copper sulfide (CuS)	1–150 UmL^−1^	0.3 UmL^−1^	[36]
Electrochemical immunosensor	gold screen-printed electrodes (AuSPEs)	1–1000 UmL^−1^	0.95 UmL^−1^	[37]
Electrochemical	aptamer-based nanobiosensor with g-C_3_N_4_/magnetic nano-structure	1–9 UmL^−1^	0.2 UmL^−1^	Current work

## Data Availability

Data will be available on request.
